# Assessing the Interactions between Zinc and Vitamin A on Intestinal Functionality, Morphology, and the Microbiome In Vivo (*Gallus gallus*)

**DOI:** 10.3390/nu15122754

**Published:** 2023-06-15

**Authors:** Cydney Jackson, Nikolai Kolba, Elad Tako

**Affiliations:** Department of Food Science, Cornell University, Ithaca, NY 14850, USA; cdj53@cornell.edu (C.J.); nk598@cornell.edu (N.K.)

**Keywords:** zinc, retinoid, vitamin A, intestine, microbiome, brush border membrane, *Gallus gallus*

## Abstract

Dietary deficiencies in zinc (Zn) and vitamin A (VA) are among the leading micronutrient deficiencies globally and previous research has proposed a notable interaction between Zn and VA physiological status. This study aimed to assess the effects of zinc and vitamin A (isolated and combined) on intestinal functionality and morphology, and the gut microbiome (*Gallus gallus*). The study included nine treatment groups (*n*~11)—no-injection (NI); H_2_O; 0.5% oil; normal zinc (40 mg/kg ZnSO_4_) (ZN); low zinc (20 mg/kg) (ZL); normal retinoid (1500 IU/kg retinyl palmitate) (RN); low retinoid (100 IU/kg) (RL); normal zinc and retinoid (40 mg/kg; 1500 IU/kg) (ZNRN); low zinc and retinoid (ZLRL) (20 mg/kg; 100 IU/kg). Samples were injected into the amniotic fluid of the fertile broiler eggs. Tissue samples were collected upon hatch to target biomarkers. ZLRL reduced ZIP4 gene expression and upregulated ZnT1 gene expression (*p* < 0.05). Duodenal surface area increased the greatest in RL compared to RN (*p* < 0.01), and ZLRL compared to ZNRN (*p* < 0.05). All nutrient treatments yielded shorter crypt depths (*p* < 0.01). Compared to the oil control, ZLRL and ZNRN reduced (*p* < 0.05) the cecal abundance of *Bifidobacterium* and *Clostridium* genera (*p* < 0.05). These results suggest a potentially improved intestinal epithelium proceeding with Zn and VA intra-amniotic administration. Intestinal functionality and gut bacteria were modulated. Further research should characterize long-term responses and the microbiome profile.

## 1. Introduction

Micronutrient deficiency is estimated to affect approximately 3–4 billion people globally—in which, 1 in 2 children and 2 in 3 women suffer from the hidden hunger for one or more essential nutrients [1,2]. Zinc deficiency (Zn) is among the leading micronutrient deficiencies globally and inadequate intake is characterized by severe consequences to host physiology and overall health, including impaired growth and development, poor epithelial maintenance, diminished cognitive function, and reduced immune response, all of which can lead to decreased infection resistance [3,4,5]. As more than 300 enzymes and transcription factors are zinc-dependent, and approximately 10% of all proteins in humans contain zinc as a cofactor, zinc plays an abundant role in biological processes, hence, severe deficiency can render the aforementioned outcomes [4,6,7]. Several animal and human studies have proposed a synergistic effect between dietary zinc and vitamin A metabolism and status [8,9,10], and it is suggested that a poor physiological status of zinc may impact vitamin A status [11]. Zinc-dependent enzyme alcohol dehydrogenase (ADH) participates in the conversion of retinol to retinal and ultimately, retinoic acid—key steps in vitamin A metabolism that are pertinent to optic function and development [10,12]. Hence, inadequate dietary zinc status is proposed to impair ADH function [13]. Further, earlier studies have observed that intra- and intercellular transport of retinol via retinol-binding protein (RBP) was zinc-dependent. A rodent model study observed that under dietary zinc-deficient conditions, retinol accumulated in the liver but was not mobilized into plasma by RBP [14]. However, zinc supplementation enabled a concomitant export of hepatic retinol and plasma vitamin A increase [14].

According to the World Health Organization, the global burden of vitamin A deficiency is a severe public health concern that primarily impacts children under the age of 5 years and women of childbearing age [15]. Poor vitamin A status is primarily characterized by blindness; however, it can also cause poor epithelial surface maintenance, reduced growth and development, impaired immune response, and increased risk of diarrhea [16,17,18]. Additionally, an inadequate zinc physiological status may potentially affect vitamin A physiological status via the microbiome [19,20]. Reed et al. (2018)’s study was the first demonstration whereby the functional capacity of a microbiome under a host zinc-deficient physiological status presented a reduced microbial capacity to metabolize retinol [19]. This study also concluded that poor zinc status unfavorably modulates the gut microbiome. Further, it is evident that populations with zinc and vitamin A deficiencies have a greater risk of diarrhea [21,22]. Although mechanisms by which dietary zinc and vitamin A nutriture interact have been postulated, these findings are not from recent years and remain limited.

Few previous studies have investigated the effects of zinc and vitamin A supplementation in order to elucidate the interrelated nature of zinc and vitamin A on host health. Regarding immune response, while Kartasurya et al. (2020) found that zinc supplementation alone improved cellular immune response in young children by enhancing interferon-gamma production, zinc supplementation after vitamin A supplementation improved the mucosal innate immune response by increasing salivary immunoglobulin A production [23]. Further, simultaneous long-term zinc and vitamin A supplementation was shown to be associated with reduced parasitic gastrointestinal infections caused by *Giardia lamblia* and *Ascaris lumbricoides* [24]. Interestingly, host physiology and development were observed to only improve significantly after the isolated supplementation of vitamin A, as revealed by the greater weight and height of preschool children [25]. There is an evident burden to readdress these nutrient interactions. Moreover, there exists a lack of robust research and biomarkers investigating intestinal functionality and the microbiome in the context of dietary zinc and vitamin A associations.

The *Gallus gallus* model is an established in vivo model that has been previously utilized to investigate the absorption and metabolism of trace minerals such as iron, calcium, and zinc [19,26,27] and non-nutritive bioactive substances [28,29]. The in vivo intra-amniotic approach has also been employed to investigate the effects of vitamin A on the immune system of chicken embryos [30]. This procedure makes use of the embryo’s amniotic fluid consumption that is naturally initiated on day 17 of embryonic development and subsequent hatch on day 21. The broiler chicken model is sensitive to dietary micronutrient deficiencies, it exhibits approximately 73% genetic homology when compared to human trace mineral and vitamin A transporters, and there is notable similarity at the gut microbial phylum level (*Bacteroidetes, Firmicutes, Proteobacteria*, and *Actinobacteria*) between broilers and humans [31,32,33]. The gut microbiome plays a pertinent role in nutrient metabolism and absorption, and studying its interactions with dietary zinc and vitamin A in broiler chickens can provide insights into potential mechanisms underlying host nutrient interactions. 

To our knowledge, this is the first study to conduct an in vivo intra-amniotic administration of combined zinc (ZnSO_4_) and vitamin A (retinyl palmitate) concentrations. ZnSO_4_ is a feed-grade form of zinc that is commonly used to supplement zinc in feed [34]; further, retinyl palmitate is the most abundant form of host vitamin A storage [35]. The objective of this study was to observe the effects of zinc and vitamin A—isolated and combined—upon intestinal functionality, duodenal morphology, and the gut microbiome within a novel system (*Gallus gallus*).

## 2. Materials and Methods

### 2.1. Zinc and Vitamin A Preparation

Zinc and vitamin A solutions were prepared as outlined in the appendix (Appendix A.1). Briefly, zinc sulfate was obtained from Beantown Chemical (Hudson, NH, USA) and retinyl palmitate was acquired from Sigma-Aldrich (CAS No.: 79–81-2; St. Louis, MO, USA). Zinc and vitamin A intra-amniotic administration concentrations were determined according to the National Research Council recommendations for broiler chicks [36]. Further, an assumed body weight of 20 g was utilized based on previously unpublished data. Zinc sulfate was diluted with 18 MΩ H_2_O to obtain a standard dose of 40 mg/kg and a marginal dose of 20 mg/kg. Retinyl palmitate was solubilized in corn oil, diluted with 18 MΩ H_2_O to obtain a 0.5% oil in water solution, and vortexed for the final vitamin A doses of 1500 IU/kg and 100 IU/kg. Vitamin A treatments were prepared in semi-dark conditions. Zinc sulfate and retinyl palmitate samples were stored in complete darkness at −20 °C until intra-amniotic administration. 

### 2.2. Animals and Design 

Cornish-cross fertile broiler chicken eggs (*n* = 100) were acquired from a commercial hatchery—Moyer’s chicks, Quakertown, PA, USA. All animal protocols were approved by Cornell University’s Institutional Animal Care and Use Committee according to the ethics approval code 2020-0077. The fertile eggs were kept incubated under constant optimal temperature and humidity in a hatchery at the Cornell University poultry farm. The osmolarity value for all injection samples was verified prior to administration to ensure a value of less than 320 Osm.

The weight of viable eggs was verified on day 17 of embryonic incubation and subsequently distributed at random into nine groups. The nine treatment groups (*n*~11) are as follows: no injection, 18 MΩ H_2_O (H_2_O only), 0.5% oil, normal ZnSO_4_ (40 mg/kg), low ZnSO_4_ (20 mg/kg), normal retinyl palmitate (1500 IU/kg), low retinyl palmitate (100 IU/kg), normal ZnSO_4_ + normal retinyl palmitate (40 mg/kg; 1500 IU/kg), and low ZnSO_4_ + low retinyl palmitate (20 mg/kg; 100 IU/kg). All treatment solutions were mixed to ensure proper content dispersion then eggs were injected with the respective treatment solution (0.5 mL) with a 21-gauge needle into the amniotic fluid (identified by candling). Immediately following, eggs were sealed with cellophane tape. On day 21, the hatchlings were weighed (Table S1) and subsequently euthanized by exposure to CO_2_. The blood, duodenum, liver, and cecum were collected and stored until further analysis.

### 2.3. Hepatic Retinol Content Quantification

Liver tissue samples were quickly weighed (15 mg) under semi-dark conditions in 2 mL tubes. 170 µL of 50% methanol (*v*/*v*) was added to each sample. A blank (no liver tissue) was processed alongside. Each sample was then homogenized and an additional 70 µL of 100% methanol was added followed by 20 µL of the internal standard (retinyl acetate (RA) (MedChemExpress, CAS No.: 127-47-9; Monmouth Junction, NJ, USA)) at 100 pmol/µL in 100% methanol. The samples were homogenized once more, vortexed at 1800 rpm for five minutes at room temperature, then incubated for an hour (−4 °C) for deproteination. Samples were centrifuged at 18,000× *g* for 10 min (4 °C), the supernatant (200 µL) was collected, then transferred into new vials in which 600 µL Methyl-tert-butyl ether (Honeywell CAT# 34875) was added. Subsequently, all samples were vortexed at 1800 rpm for 10 min (room temperature). The upper organic phase was transferred into a clean glass culture tube and evaporated to dryness in a speed vac. The extract was reconstituted into 200 µL of 75% methanol (*v*/*v*).

The prepared samples were measured using high-performance liquid chromatography–mass spectrometry (HPLC-MS). The HPLC-MS apparatus (ExionLC, Framingham, MA, USA) was used with a C8 column (Restek, Bellefonte, PA, USA; 100 mm × 1.0 mm) with a 50 µL autoinjector loop. Mobile phase A: H_2_O/0.1% formic acid; mobile phase B: 100% methanol. The total run time was 30 min at a constant flow rate (100 µL/min) and temperature (5 °C) with the following gradient program: 0–2 min to 20% A, 80% B; 2–5 min holding at 20% A, 80% B; 5–17 min to 2% A, 98% B; 17–23 min holding at 2% A, 98% B; 23–25 min to 20% A, 80% B; 25–30 min holding at 20% A, 80% B. The Sciex OS 2.0 software was used for quantitation analysis.

### 2.4. Plasma Zinc Content Analysis

On the day of the hatch, blood was collected from the heart and placed into micro-hematocrit heparinized capillary tubes (Fisher Scientific Waltham, MA, USA). Serum zinc content was quantified using an inductively coupled argon-plasma/atomic emission spectrophotometer.

### 2.5. Total RNA Extraction

Total RNA extraction was completed with liver and duodenal tissue samples (30 mg) using the Qiagen RNeasy Mini Kit (RNeasy Mini Kit, Qiagen Inc., Valencia, CA, USA) according to the manufacturer’s protocol. Briefly, buffer RLT with β-Mercaptoethanol was added to the samples prior to homogenization. After disrupting the tissue, the samples were centrifuged, and the supernatant was collected. The remaining lysate was washed with ethanol, buffer RW1, and buffer RPE (twice) while undergoing centrifugation between each buffer wash. Finally, the samples were treated with RNase-free water and centrifuged for one minute at 8000× *g* to elute the RNA. The remaining content was stored at −20 °C until used.

### 2.6. Reverse Transcriptase Polymerase Chain Reaction (RT-PCR)

A 20 µL reverse transcriptase reaction was used to create the cDNA from the extracted RNA. To complete the reaction, the BioRad C1000 touch thermocycler (Bio-Rad, Hercules, CA, USA) using the Improm-II Reverse Transcriptase Kit (CAT# A1250; Promega, Madison, WI, USA) was utilized. The cDNA concentration was measured by Nanodrop (Thermo Fisher Scientific, Waltham, MA, USA) at an absorbance of 260 nm and 280 nm using an extinction coefficient of 33 (for single-stranded DNA). Genomic DNA contamination was assessed by a real-time RT-PCR assay for the reference gene samples.

The primers used in RT-PCR were designed based on relevant gene sequences from the GenBank database using the Real-Time Primer Design Tool software (IDT DNA, Coralville, IA, USA). Table 1 depicts the primer sequences relevant to zinc and vitamin A metabolism, immune response, and brush border membrane functionality, with the *Gallus gallus* primer 18S rRNA as the reference gene. Primer specificity was verified through BLAST searches against the genomic National Center for Biotechnology Information (NCBI) database.

### 2.7. Microbial Samples and Intestinal Contents DNA Isolation

All protocols were conducted as previously reported [37,38]. Briefly, the cecum contents were isolated and placed into 15 mL tubes containing PBS (pH 7.4) under sterilized conditions. Glass beads (4-mm diameter) were added, and the samples were vortexed for three minutes and immediately centrifuged at 1000× *g* for five minutes. The supernatant was collected and centrifuged at 4000× *g* for 10 min. The pellet was washed with PBS and stored at −20 °C until DNA purification. DNA extraction was conducted by treating the pellet with 50 mM EDTA and 10 mg/mL lysozyme at 37 °C. Ultimately, DNA purification was completed by Wizard Genomic DNA purification kit according to the manufacturer’s protocol (Promega Corp., Madison, WI, USA).

### 2.8. Primer Design and PCR Amplification of Bacterial 16S rDNA

The following primers were used: *Bifidobacterium*, *Lactobacillus*, *Escherichia coli*, *Clostridium*, and 16S rDNA (universal) according to what was previously described [39,40,41]. Essentially, each bacterial group is reported as a relative proportion of the bacteria to the universal primer. PCR products were applied to 1.5% agarose gel with ethidium bromide stain and quantified with Gel-Pro analyzer version 3.0 (Media Cybernetics LP, Rockville, MD, USA).

### 2.9. Histomorphological Examination

Proximal duodenal tissues were collected on the day of hatch and immediately placed into 4% (*v*/*v*) buffered formaldehyde and stored at room temperature, as described previously [38,42,43]. Later, the samples were dehydrated, cleared, and embedded into paraffin. Sections were sliced (5 µM), placed on glass slides where duodenal sections were deparaffinized in xylene and rehydrated in graded alcohol, and ultimately stained (Alcian Blue/Periodic acid-Schiff). Histomorphological assessment commenced by using light microscopy and relevant software (EPIX XCAP—Standard version, Olympus, Waltham, MA, USA). For each treatment group, three biological samples (*n* = 3) with six segments each were measured and analyzed. Villi, crypts, and cell measurements and counts were performed by selecting ten of each at random per segment. To calculate villus surface area, the following equation was used:

$$Villus\;surface\;area=2\pi \times \frac{VW}{2}\times VL$$

where *VW* is the average of three villus width measurements, and *VL* is the villus length.

### 2.10. Statistical Analysis

Values are reported as the means ± standard error mean (SEM). All parameters were tested for normal distribution and equal variance using a Shapiro-Wilk test. If accepted, a one-way analysis of variance (ANOVA) was utilized followed by a Tukey post-hoc test to identify significance based on *p* < −0.05. Non-normally distributed parameters were analyzed using the Kruskal–Wallis test followed by Dunn’s post-hoc test (*p* < 0.05 or *p* < 0.01). Statistical analyses were conducted using GraphPad Prism (version 8.0) and R Studio (version 2022.12.0+353). 

## 3. Results

### 3.1. Plasma Zinc and Liver Retinol Content

Table 2 depicts the plasma zinc and liver retinol concentrations. Between all experimental groups, there were no significant differences observed for concentrations of plasma zinc, nor were differences observed for hepatic retinol levels.

### 3.2. Gene Expression of Duodenal and Hepatic Zinc-Relevant Metabolism Proteins

**Figure 1:**
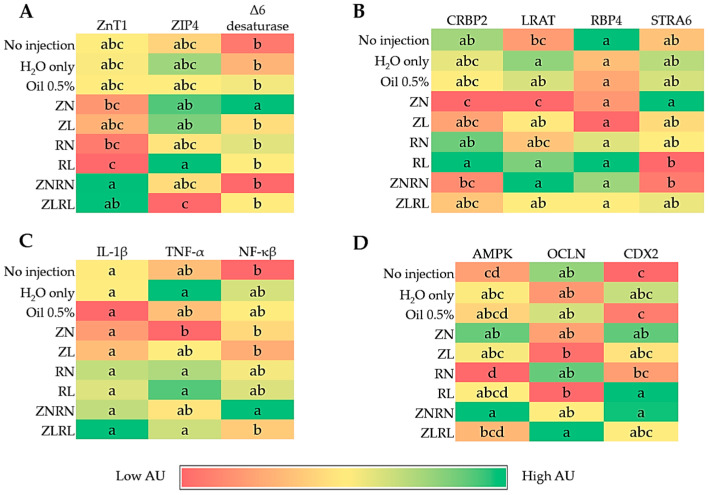
Effect of intra-amniotic administration of zinc and vitamin A on zinc (**A**), vitamin A (**B**), inflammatory (**C**), and functional (**D**) protein gene expression duodenal and hepatic (Δ6 desaturase, RBP4, and STRA6). Values are the means ± SEM, *n* = 3–5. ^a–d^ Treatment groups not indicated by the same letter in the same column are significantly different (*p* < 0.05) by Kruskal–Wallis with Dunn’s post-hoc test (ZnT1, ZIP4, and OCLN) or ANOVA with Tukey post-hoc test (Δ6 desaturase, CRBP2, LRAT, RBP4, STRA6, IL-1β, TNF-α, NF-κB, AMPK, and CDX2). ZnT1, Zinc transporter 1; ZIP4, Zinc transporter 4; CRBP2, Cellular retinol-binding protein 2; LRAT, Lecithin/Retinol Acyltransferase; RBP4, Retinol binding protein 4; STRA6, Stimulated by Retinoic aid 6; NF-κB, Nuclear factor kappa beta; TNF-α, Tumor necrosis factor-alpha; IL-1β, Interleukin beta; AMPK, AMP-activated kinase protein; OCLN, Occludin; SI, Sucrase isomaltase; CDX2, Caudal-type homeobox 2.

Figure 1A depicts the impacts of zinc and vitamin A treatments on the gene expression of proteins related to zinc and its metabolism. The greatest (*p* < 0.05) expression of ZnT1, a protein located on the duodenal basolateral membrane, was observed in the ZNRN and ZLRL groups respectively when compared to group RL which was the lowest gene expression of ZnT1. ZIP4 gene expression was lowest (*p* < 0.05) in the ZLRL group when compared to RL, ZL, and ZN treatment groups. The gene expression of Δ6 desaturase within the liver was highest (*p* < 0.05) in the ZN group compared to all other treatments, where every group excluding ZN was significantly similar.

### 3.3. Gene Expression of Duodenal and Hepatic Retinoid-Related Metabolism Proteins

Duodenal retinoid metabolism protein gene expression was investigated in CRBP2 and LRAT Figure 1B. The greatest (*p* < 0.05) increase in CRBP2 gene expression was evident in the RL group when compared to ZN and ZNRN treatments. Yet, the gene expression of CRBP2 in the RL group was similar to the no injection, H_2_O only and Oil 0.5% controls, the RN group, and the ZLRL group. Moreover, the gene expression of LRAT was significantly lowered in the ZN treatment group compared to the zinc and vitamin A treatment groups. Treatment groups administered vitamin A (RN, RL, ZLRL, ZNRN) had similar LRAT gene expression. Gene expression of hepatic RBP4 was similar throughout all experimental groups. However, hepatic STRA6 gene expression was greatest (*p* < 0.05) in the ZN group when compared to the RL and ZNRN treatments. Yet, when comparing the controls (no injection, H_2_O only, and Oil 0.5%) to all treatment groups, significant differences were not evident.

### 3.4. Gene Expression of Duodenal Inflammatory-Related Proteins 

There were no differences observed for duodenal IL-1β gene expression between all experimental groups Figure 1C. The gene expression of TNF-α was lowered significantly in the ZN group as compared to the H_2_O control, and RN, RL, and ZLRL treatments. All other treatment groups (excluding ZN) were similar to the controls. The highest (*p* < 0.05) upregulation of NF-κB was in the ZNRN group, whereas ZLRL, ZL, ZN, and no injection groups were lower (*p* < 0.05) than ZNRN, yet similar to the remaining controls (H_2_O only and Oil 0.5%) and vitamin A-treated groups.

### 3.5. Gene Expression of Duodenal Functionality-Relevant Proteins

The gene expression of brush border membrane functionality proteins varied throughout treatment groups. AMP-activated kinase (AMPK) protein gene expression increase (*p* < 0.05) was evident in the ZNRN treatment when compared to the ZLRL group. There was an upregulation (*p* < 0.05) of occludin (OCLN) protein gene expression in the ZLRL group as compared to ZL and RL treatments, but no difference was observed when compared to the control groups. The gene expression of Caudal-type homeobox 2 (CDX2) was significantly lowered in the RN treatment when compared to RL, but similar between each concentration of zinc and each treatment administered zinc and vitamin A. Overall CDX2 gene expression was greatest (*p* < 0.05) in ZNRN, RL, and ZN when compared to the no injection and Oil 0.5% controls.

### 3.6. Duodenal Morphology

**Figure 2:**
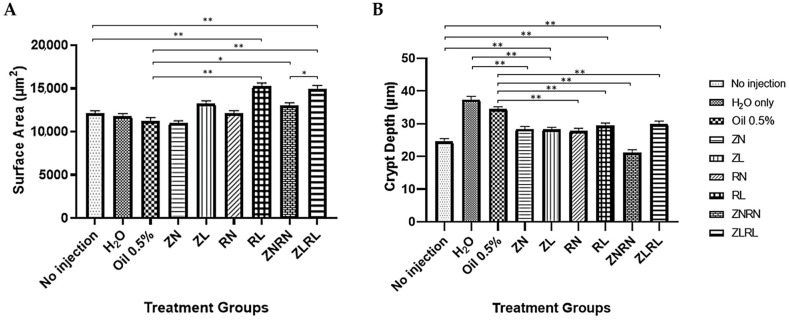
Effect of the intra-amniotic administration of zinc and vitamin A on duodenal villi surface area and crypt depth. Bars are the means ± SEM, *n* = 3 animals/group, 6 sections, 10 measurements, with significance (* = *p* < 0.05; ** = *p* < 0.01) based on Kruskal–Wallis followed by a post-hoc Dunn’s test. See supplemental material (Tables S2 and S3) for statistical significance between experimental groups. (**A**) Duodenal villi surface area (µm^2^); (**B**) Duodenal crypt depth (µm).

Duodenal villi surface area (Figure 2A) was significantly greater in the RL group when compared to the no injection and Oil 0.5% controls (*p* < 0.01). Villi surface area was also increased significantly in the ZNRN group when compared to the Oil 0.5% control (*p* < 0.05). Further, the ZLRL treatment was greater than the no injection and Oil 0.5% controls (*p* < 0.01), and the ZNRN treatment group (*p* < 0.05). Moreover, ZN and ZL group crypt depth was shorter (*p* < 0.05) than the H_2_O-only group, and ZL was also shorter (*p* < 0.05) than the no injection control. RL group crypt depth was observably smaller (*p* < 0.05) than the no injection control, and both RL and RN groups had a shorter (*p* < 0.05) crypt depth than the Oil 0.5% control. Crypt depth in the ZNRN group was shorter (*p* < 0.01) than the Oil 0.5% control, and the ZLRL group crypt depth was significantly shorter than the Oil 0.5% control. 

The villi goblet diameters of ZLRL and ZNRN were the greatest among all of the treatment and control groups, as shown in Table 3. Group RL villi goblet diameter was greater (*p* < 0.05) than group RN and the controls except for the no-injection control which was similar. No differences were observed between the zinc-only treatments, yet ZN and ZL were also among the significantly lowest villi goblet cell diameter measurements. The largest (*p* < 0.05) diameter for goblet cells located in the crypt was observed in the ZNRN group when compared to the ZLRL treatment, the zinc-only and RN treatments, and the controls. Within the vitamin A-only treatments, group RL crypt goblet cell diameter was greater (*p* < 0.05) than the RN group and the controls, while there were no differences observed between the zinc-only treatments and controls. Moreover, there was a higher (*p* < 0.05) count of goblet cells in the crypt per unit area in the RN group when compared to all other treatments, but not when compared to the RL group on no injection control (*p* > 0.05). The lowest (*p* < 0.05) crypt goblet cell number count was observed in treatment groups ZN and ZLRL—in which the ZN group was significantly lower than ZL and the control groups, and ZLRL was lower (*p* < 0.05) than the ZNRN group and the controls.

Although the abundance of acidic goblet cells per unit area was greatest in the RN group among treatment groups, significance is only observed when compared to ZN and ZLRL treatments and the Oil 0.5% control. Neutral goblet cell count within the crypt per unit area was increased significantly in the ZLRL group compared to the ZNRN group and all control groups. The abundance of mixed goblet cells was lowered significantly in the RN and RL groups as well as in the ZNRN and ZLRL groups relative to the no injection and Oil 0.5% controls.

Table 4 depicts the observed effects of zinc and vitamin A on Paneth cells in the duodenal crypts. Group ZN had the greatest (*p* < 0.05) count of Paneth cells per unit area when compared to No injection and H_2_O-only controls, as well as RN, ZNRN, and ZLRL treatments. Among treatment groups, the largest Paneth cell diameter was observed in ZN, RN, and ZLRL, whereas group ZL had the smallest Paneth cell diameter.

### 3.7. Cecal Bacterial Abundance of Select Populations

**Figure 3:**
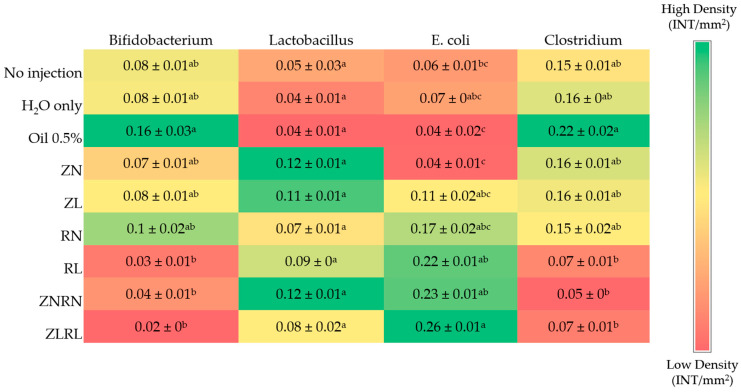
Effect of intra-amniotic administration of zinc and vitamin A on select cecal bacterial (genera- or species-level) populations. Log transformed values are the means ± SEM, *n* = 5. ^a–c^ Treatment groups not indicated by the same letter in the same column are significantly different (*p* < 0.05) by Kruskal–Wallis followed by a Dunn’s test.

The relative abundance of *Bifidobacterium* was greatest (*p* < 0.05) in the Oil 0.5% control group, while the lowest abundance was observed in treatment groups RL, ZNRN, and ZLRL (Figure 3). There were no significant differences between treatment groups and controls for *Lactobacillus* abundance in the cecum. The ZLRL group had a great abundance of E. coli compared to No injection and Oil 0.5% controls and the ZN group. Further, the abundance of *E. coli* in the ZNRN and RL groups was significantly greater than the Oil 0.05% control and ZN group. Lastly, the relative abundance of *Clostridium* was reduced in RL, ZNRN, and ZLRL groups compared to Oil 0.05% control.

## 4. Discussion

Given that there is a lack of research assessing intestinal functionality and the microbiome in the context of dietary zinc and vitamin A associations, this present study was conducted to investigate the isolated and combined effects of zinc and vitamin A nutriture via intra-amniotic administration. Intra-amniotic administration is a method that has been previously used to investigate the effects of particular nutrients on host physiology [41,43,44,45]. The broiler chicken is a valuable model for investigating the associations between dietary zinc and vitamin A on host physiology due to the genetic homology with human nutrient metabolism proteins, taxonomic similarity at the phylum level exists, and sensitivity to micronutrient deficiencies [31,32,33].

Concerning gene expression, mRNA expression was assessed in duodenal and hepatic tissue for zinc, vitamin A, and inflammatory- and functionality-related proteins. From the SLC39 family, ZIP4 is a zinc influx transport protein located on the apical side of the brush border membrane that imports zinc into the cytoplasm of the enterocyte [6,46]. A recent review by Kambe et al. (2015) thoroughly highlighted the unique role of ZIP4, as it is only expressed during dietary zinc deficiency [6]. In our study, we observed that the marginal concentration of zinc and vitamin A combined significantly (*p* < 0.05) downregulated the expression of ZIP4 when compared to RL, ZL, and ZN treatment groups (Figure 1A). This suggests an improved level of zinc in duodenal tissue despite the marginal dietary concentrations of zinc and vitamin A administered. This may be further supported by the observed upregulation of ZnT1 mRNA expression in the combined zinc and vitamin A treatments. ZnT1 is the zinc efflux transporter protein located on the basolateral membrane of the enterocyte that functions to shuttle cytosolic zinc to the blood for systemic distribution [47,48]. Upregulation of ZnT1 mRNA occurs due to increased cellular zinc [6,49]. Although plasma zinc concentrations did not differ between treatments (Table 2) and Δ6 desaturase gene expression was only upregulated in the ZN group, we hypothesize the aforementioned results suggest an initial enhanced metabolic response within the primary site of absorption for zinc due to the presence of vitamin A [27]. Hence, we believe a longer exposure to the nutrients would produce a significant systemic response. For instance, a six-week feeding trial conducted in broiler chickens by Knez et al. (2018) observed an upregulation of Δ6 desaturase gene expression, higher serum zinc concentration, and lower LA:DGLA (linoleic acid/dihomo-γ-linolenic acid) ratio in treatments administered high-zinc biofortified wheat [50]. Moreover, Figure 1B reveals the gene expression of vitamin A intestinal (CRBP2 and LRAT) and hepatic (RBP4 and STRA6) metabolism proteins. Likewise with zinc, hepatic retinol concentrations did not differ; yet CRBP2 and LRAT gene expression significantly differed among treatment groups. Enterocytes contain CRBP2 (cellular retinol-binding protein-II)—a retinol-binding protein that is expressed to deliver retinol to LRAT (lecithin:retinol acyltransferase) whereby retinol is esterified to form retinol ester for further transportation and hepatic storage [51,52]. Few studies have investigated the impact of vitamin A status on duodenal CRBP2 and LRAT gene expression, and our findings reveal an upregulation of LRAT when the marginal concentration of vitamin A (RL) was administered and a significant downregulation when the adequate concentration of zinc (ZN) was administered. Previous research has reported that levels of LRAT gene expression decrease in the heart, liver, and lungs of vitamin A-deficient animals [53,54,55]. Our findings indicate that the administration of zinc alone did not suggest an improved vitamin A status via vitamin A metabolism protein gene expression. Hepatic expression of RBP4 (retinol-binding protein 4) and STRA6 (Stimulated by retinoic acid 6) did not differ when compared to the no injection control. RBP4 functions to export retinol from the liver to extrahepatic tissues, while STRA6 is a transmembrane transporter of the retinol-RBP4 complex [56,57,58]. We believe a longer exposure to the treatments would stimulate a clearer gene expression of hepatic vitamin A metabolism proteins. 

Moreover, vitamin A is known for its maintenance role in gut homeostasis via immune response and cell differentiation [59]. IL-1β is a proinflammatory cytokine that initiates an inflammatory response, and as such, the similarity throughout all treatments and the subsequent inflammatory response elements (TNF-α and NF-κB) indicates the absence of duodenal epithelial inflammation [60,61]. Further, our results reveal the ZN and ZNRN treatments simultaneously elevate AMPK and CDX2 expression. AMPK participates in maintaining intestinal epithelial integrity via tight junctions [62], however, occludin expression did not differ in the aforementioned groups. Since zinc exposure is also hypothesized to induce AMPK activation [62,63], this may have influenced AMPK upregulation. Conversely, while a previous study by Kim et al. (2015) found retinoic acid supplementation in human endothelial cells increased AMPK phosphorylation [64], our results do not agree. Therefore, future studies should include utilizing the active metabolite, retinoic acid, to observe the effects on gastrointestinal functionality. We observed a concomitant upregulation of AMPK and CDX2 in the normal zinc and normal zinc with vitamin A treatments. CDX2, or caudal-type homeobox 2, is a transcription factor that AMPK promotes to enhance intestinal epithelial homeostasis by regulating cell differentiation [65].

Although zinc and vitamin A are individually known for their maintenance role in gastrointestinal homeostasis, the interactions between vitamin A and zinc on intestinal epithelial morphology are not well characterized [66,67]. Interestingly, marginal combined vitamin A and zinc treatments enhanced the villi surface area greater than the normally combined treatments (See Figure S1 for representative duodenal morphometric images). A similar trend was also observed as the marginal treatment of isolated vitamin A improved villi surface area greater than the normal vitamin A treatment. A study by Wang et al. (2020) reported long-term vitamin A supplementation in piglets increased jejunal villus height and surface area by regulating intestinal stem cells [68]. However, the study also observed an increased crypt depth which was contrary to our results (Figure 2B). All treatments of zinc, vitamin A, and the nutrients combined yielded shorter crypt depths (*p <* 0.01) when compared to the water or 0.5% oil controls. Intestinal morphology is a primary indicator of gastrointestinal development and health— particularly, villus structure and crypt depth [69,70]. The lengthening of villi and shortened crypt depth in broilers was previously associated with sufficient growth performance and increased nutrient metabolism [71]. Indeed, enterocytes emerge from the crypts of Lieberkühn and migrate onto the villus to facilitate nutrient metabolism and absorption [72]. The shorter crypts and larger surface area are indicative of enhanced small intestine health because a slower enteric epithelial cell turnover rate allows sufficient time for cellular differentiation and therefore, optimal enterocyte function [73]. Overall, we observed a synergistic interaction between zinc and vitamin A for intestinal epithelial maintenance: increased villi goblet cell diameter in ZLRL and ZNRN groups, and increased crypt goblet cell diameter in group ZNRN. Further, group ZLRL reduced the number of goblet cells per unit area within the crypts. Goblet cells are endogenous to intestinal epithelial crypts with specialized functions to support the enteric environment via mucin glycoproteins and mucous production [74]. Poor vitamin A physiological status was previously shown to increase the number of goblet cells and cause severe atrophy [75]. While utilizing a murine model, the authors also reported decreased Paneth cell count and overall dysfunction of intestinal epithelial cells. We did not observe a similar trend in our study (Table 4) perhaps due to the one-time administration of the treatments, whereas the previous study was conducted with an aging model over an extended period.

The complex concomitance of gut microorganisms and nutrients is essential for host physiology [67,76,77]. Hence, we assessed the relative abundance of select cecal microbial populations after zinc and vitamin A doses (Figure 3). When compared to the 0.5% oil control, we found marginal VA administration and both marginal and normal administration of combined Zn/VA to decrease the abundance of *Bifidobacterium* and *Clostridium* genera yet increase the relative abundance of *E. coli*. Previous work in various animal models has reported Zn and VA to alter gut microbiota; however, the findings are often inconsistent. A recent study revealed ZnSO_4_ supplementation increased total gut bacteria in weaned piglets, but without modulation of *Lactobacillus*, *E. coli*, and *Bifidobacterium* abundance [78]. However, in a study using a pigeon squab model, the authors reported 10 mg daily supplementation of zinc methionine reduced the abundance of *Lactobacillus*, *Enterococcus,* and *Bifidobacterium* populations [79]. Host age is also a factor that can impact the microbiome and must be considered when interpreting results: Davis et al., (2022) found *Lactobacillus* species to increase in Zn-supplemented older mice, but not in young mice [80]. It appears that in the study age-related effects contributed to beta diversity more than dietary zinc status which was correlated to immunomodulatory-related taxa such as *Lactobacillus* spp. and *Ruminococcaceae* spp. Moreover, VA supplementation was shown to increase the abundance of *Bifidobacterium* spp. in VA-supplemented male infants. However, this was not observed in VA-supplemented female infants [81]. Further, the complex interactions between VA and gut microbiota were elucidated to contribute to gut homeostasis via commensal bacteria such as *Lactobacillus* spp. [82] and *Bifidobacterium bifidum* [83] independently converting dietary retinol to retinoic acid (the active form of VA). This conversion in the intestinal mucus layer can ultimately influence host physiology as retinoic acid uptake occurs into enterocytes, regulates gene expression through retinoic acid response elements, and mediates intestinal immune response [83,84].

## 5. Conclusions

Here, we have assessed the effects of Zn, VA, and combined Zn/VA on intestinal morphology, functionality, and the microbiome utilizing a novel, naïve in vivo model. Overall, these results suggest a potentially improved intestinal epithelium proceeding with Zn and VA intra-amniotic administration. Intestinal functionality and gut bacteria were modulated, albeit differently than in previous studies. Moreover, the intra-amniotic administration approach is an advantage of this study in that a physiological response was garnered in a living, developing system without requiring the use of a diet. Further research and characterization of long-term responses and the gut microbiome profile should be completed.

## Figures and Tables

**Table 1:** DNA primers.

Analyte	Forward Primer (5′-3′)	Reverse Primer (5′-3′)	Base Pair	GI Identifier
Zinc-Related				
ZnT1	GGTAACAGAGCTGCCTTAACT	GGTAACAGAGCTGCCTTAACT	105	54109718
ZIP4	TCTCCTTAGCAGACAATTGAG	GTGACAAACAAGTAGGCGAAAC	95	107050877
Δ6 desaturase	GGCGAAAGTCAGCCTATTGA	AGGTGGGAAGATGAGGAAGA	93	261865208
Vitamin A Metabolism				
CRBP2	GGCTACATGGTTGCACTAGACA	AACCACCCGGTTATCGAGTC	195	NM_001277417.1
LRAT	GATTTTGCCTATGGCGGCAG	TTGTCGGTCTGGAAGCTGAC	197	XM_420371.7
RBP4	TGCCACCAACACAGAACTCTC	CTTTGAAGCTGCTCACACGG	149	NM_205238.2
STRA6	GTGCGCTGAACTTTGTCTGC	TTCTTCCTGCTCCCGACCT	116	NM_001293202.2
Inflammatory Response				
NF-κB	CACAGCTGGAGGGAAGTAAAT	TTGAGTAAGGAAGTGAGGTTGAG	100	2130627
TNF-α	GACAGCCTATGCCAACAAGTA	TTACAGGAAGGGCAACTCATC	109	53854909
IL-1β	CTCACAGTCCTTCGACATCTTC	TGTTGAGCCTCACTTTCTGG	119	88702685
Brush Border Membrane Functionality				
AMPK	CTCCACTTCCAGAAGGTTACTT	GCAGTAGCTATCGTTCATCCTATC	140	427185
OCLN	GTCTGTGGGTTCCTCATCGT	GTTCTTCACCCACTCCTCCA	124	396026
CDX2	CCAGCAATGCCAGCATATTG	CGGTTTCTCCTTACCACTTCTT	95	2246388
18S rRNA	GCAAGACGAACTAAAGCGAAAG	TCGGAACTACGACGGTATCT	100	7262899

ZnT1, Zinc transporter 1; ZIP4, Zinc transporter 4; CRBP2, Cellular retinol-binding protein 2; LRAT, Lecithin/Retinol Acyltransferase; RBP4, Retinol binding protein 4; STRA6, Stimulated by Retinoic aid 6; NF-κB, Nuclear factor kappa beta; TNF-α, Tumor necrosis factor-alpha; IL-1β, Interleukin beta; AMPK, AMP-activated kinase protein; OCLN, Occludin; SI, Sucrase isomaltase; CDX2, Caudal type homeobox 2.

**Table 2:** Effect of the intra-amniotic administration of zinc and vitamin A on plasma zinc (µg/mL) and liver retinol content (pmol/mg).

Treatment Group	No Injection	H_2_O Only	Oil 0.5%	ZN	ZL	RN	RL	ZNRN	ZLRL
Plasma zinc (µg/mL)	0.700 ± 0.322 ^a^	0.471 ± 0.006 ^a^	0.518 ± 0.113 ^a^	0.609 ± 0.067 ^a^	0.756 ± 0.101 ^a^	0.343 ± 0.037 ^a^	0.507 ± 0.118 ^a^	0.578 ± 0.058 ^a^	0.619 ± 0.116 ^a^
Liver retinol (pmol/mg)	16.099 ± 0.527 ^a^	16.605 ± 2.518 ^a^	19.020 ± 0.389 ^a^	18.234 ± 1.529 ^a^	17.023 ± 1.328 ^a^	26.590 ± 5.153 ^a^	21.467 ± 3.665 ^a^	21.577 ± 1.561 ^a^	19.226 ± 1.094 ^a^

Values are the means ± SEM, *n* = 3. ^a^ Treatment groups not indicated by the same letter in the same row are significantly different (*p* < 0.05) by Kruskal–Wallis with Dunn’s post-hoc test.

**Table 3:** Effect of the intra-amniotic administration of zinc and vitamin A on goblet cells located on duodenal villi and crypt.

Treatment Group	Villi Goblet Diameter (µm)	Crypt Goblet Diameter (µm)	Crypt Goblet Cell Number	Crypt Goblet Cell Type		
				Acidic	Neutral	Mixed
No injection	3.37 ± 0.06 ^bc^	2.92 ± 0.06 ^d^	6.33 ± 0.25 ^ab^	5.52 ± 0.23 ^abc^	0.01 ± 0.01 ^d^	0.84 ± 0.08 ^a^
H_2_O only	3.15 ± 0.07 ^cd^	3.04 ± 0.07 ^cd^	7.11 ± 0.25 ^a^	6.59 ± 0.25 ^a^	0.01 ± 0.01 ^d^	0.51 ± 0.06 ^abc^
Oil 0.5%	2.83 ± 0.07 ^d^	2.90 ± 0.07 ^d^	4.53 ± 0.19 ^c^	3.84 ± 0.19 ^e^	0.07 ± 0.02 ^bcd^	0.62 ± 0.06 ^ab^
ZN	2.93 ± 0.07 ^d^	2.79 ± 0.07 ^d^	3.23 ± 0.14 ^d^	2.60 ± 0.14 ^f^	0.15 ± 0.03 ^bc^	0.55 ± 0.07 ^bc^
ZL	3.03 ± 0.07 ^d^	2.80 ± 0.06 ^d^	4.64 ± 0.22 ^c^	4.40 ± 0.35 ^de^	0.07 ± 0.03 ^cd^	0.27 ± 0.04 ^c^
RN	2.90 ± 0.07 ^d^	3.09 ± 0.07 ^cd^	5.91 ± 0.26 ^ab^	5.40 ± 0.25 ^abcd^	0.08 ± 0.02 ^bcd^	0.55 ± 0.08 ^bc^
RL	3.70 ± 0.08 ^b^	3.58 ± 0.07 ^ab^	5.51 ± 0.23 ^bc^	5.01 ± 0.23 ^bcd^	0.17 ± 0.03 ^ab^	0.33 ± 0.05 ^c^
ZNRN	4.12 ± 0.08 ^a^	3.65 ± 0.05 ^a^	5.28 ± 0.23 ^bc^	4.82 ± 0.23 ^bcde^	0.09 ± 0.03 ^bcd^	0.41 ± 0.06 ^c^
ZLRL	4.08 ± 0.07 ^a^	3.29 ± 0.06 ^bc^	3.15 ± 0.16 ^d^	2.39 ± 0.16 ^f^	0.32 ± 0.05 ^a^	0.51 ± 0.06 ^bc^

Values are the means ± SEM, *n* = 5. ^a–f^ Treatment groups not indicated by the same letter in the same column are significantly different (*p* < 0.05) by Kruskal–Wallis with Dunn’s post-hoc test.

**Table 4:** Effect of the intra-amniotic administration of zinc and vitamin A on Paneth cells.

Treatment Group	No Injection	H_2_O Only	Oil 0.5%	ZN	ZL	RN	RL	ZNRN	ZLRL
Crypt Paneth Cell Number	1.17 ± 0.06 ^de^	1.31 ± 0.04 ^bcd^	1.36 ± 0.05 ^abcd^	1.49 ± 0.05 ^a^	1.32 ± 0.04 ^abc^	1.07 ± 0.02 ^e^	1.38 ± 0.04 ^ab^	1.23 ± 0.03 ^bcde^	1.18 ± 0.03 ^de^
Paneth Cell Diameter (µm)	1.69 ± 0.03 ^a^	1.56 ± 0.03 ^abc^	1.46 ± 0.03 ^cde^	1.64 ± 0.03 ^a^	1.33 ± 0.02 ^e^	1.59 ± 0.03 ^ab^	1.41 ± 0.03 ^de^	1.47 ± 0.02 ^bcd^	1.59 ± 0.03 ^ab^

Values are the means ± SEM, *n* = 5. ^a–e^ Treatment groups not indicated by the same letter in the same column are significantly different (*p* < 0.05) by Kruskal–Wallis with Dunn’s post-hoc test.

## Data Availability

Not applicable.

## Supplementary Materials

The following supporting information can be downloaded at: https://www.mdpi.com/article/10.3390/nu15122754/s1, Table S1: Effect of the intra-amniotic administration of zinc and vitamin A on bodyweight (g); Table S2: Dunn’s post-hoc test on duodenal villi surface area data; Table S3: Dunn’s post-hoc test on duodenal crypt depth data; Figure S1: Representative images of duodenal morphology per treatment.

## Appendix A

### Appendix A.1. Zinc and Vitamin A Preparation

Zinc sulfate (ZnSO_4_) was obtained from Beantown Chemical (Hudson, NH, USA) and retinyl palmitate (RP) was acquired from Sigma-Aldrich (CAS No.: 79-81-2; St. Louis, MO, USA). ZnSO_4_ and RP intra-amniotic administration concentrations were determined according to the National Research Council recommendations for broiler chicks [36]. An assumed bodyweight of 20 g was utilized based on previous unpublished data. Stock solutions were prepared by diluting ZnSO_4_ with 18 MΩ H_2_O (10 mg/mL) and RP with corn oil (10 mg/mL). Then, the solution for normal ZnSO_4_ treatment was prepared by diluting 1.6 mL of the ZnSO_4_ stock in 8.4 mL 18 MΩ H_2_O, and the low ZnSO_4_ treatment solution was prepared by diluting 0.8 mL of the ZnSO_4_ stock in 9.2 mL 18 MΩ H_2_O. Therefore, ZnSO_4_ was diluted with 18 MΩ H_2_O to obtain a standard dose of 40 mg/kg and a marginal dose of 20 mg/kg.

Further, normal RP solution was prepared by diluting 33 µL of RP stock solution in 17 µL corn oil and 9.95 mL of 18 MΩ H_2_O, and the low RP solution was prepared by diluting 2.2 µL of RP stock solution in 47.8 µL corn oil and 9.95 mL of 18 MΩ H_2_O. Overall, RP was solubilized in corn oil and diluted with 18 MΩ H_2_O to obtain a 0.5% oil in water solution for the final doses of 1500 IU/kg and 100 IU/kg.

In addition, the treatment of normal ZnSO_4_ and RP was prepared by adding 1.59 mL of the ZnSO_4_ stock to 7.91 mL 18 MΩ H_2_O, then 33 µL of the RP stock and 17 µL of corn oil was added. Lastly, the treatment of low ZnSO_4_ and RP was prepared by adding 0.796 mL of the ZnSO_4_ stock to 8.704 mL 18 MΩ H_2_O, then 2.2 µL of the RP stock and 47.8 µL of corn oil was added. Essentially, the solutions were prepared as combined doses to obtain a normal zinc and vitamin A combined solution (40 mg/kg; 1500 IU/kg) and a marginal zinc and vitamin A combined solution (20 mg/kg; 100 IU/kg). All solutions containing vitamin A were prepared in semi-dark conditions. Solutions were vortexed and stored in completed darkness at −20 °C until intra-amniotic administration.
