# Supplementary material for: Assessing the Interactions between Zinc and Vitamin A on Intestinal Functionality, Morphology, and the Microbiome In Vivo (Gallus gallus)
Source: Nutrients. 2023 Jun 15;15(12):2754. doi: 10.3390/nu15122754 (PMC10302570; doi:10.3390/nu15122754)
Supplement: Supplementary file 1 [file nutrients-15-02754-s001.zip › nutrients-2440095-supplementary.pdf]

1. Bodyweight

Table S1. Effect of the intra-amniotic administration of zinc and vitamin A on bodyweight (g).

Treatment Group	No injection	H_2O only	Oil 0.5%	ZN	ZL	RN	RL	ZNRN	ZLRL
Bodyweight (g)	41.67 ± 1.34[a]	43.21 ± 1.36[a]	40.31 ± 1.56[a]	39.63 ± 1.93[a]	40.48 ± 1.51[a]	41.45 ± 1.02[a]	42.62 ± 1.21[a]	41.45 ± 1.18[a]	40.90 ± 0.6[a]

Values are the means ± SEM, n = ~11. Treatment groups not indicated by the same letter in the same row are significantly different ($p < 0.05$) by ANOVA with Tukey post-hoc test.

2. Duodenal Villi Surface Area Kruskal-Wallis Test with Dunn's Post-hoc Test

Kruskal-Wallis test
P value: <0.0001
Exact or approximate P value?: Approximate
P value summary: ****
Do the medians vary significantly (P < 0.05)?: Yes
Number of groups: 9
Kruskal-Wallis statistic: 129.5

Data summary
Number of treatments (columns): 9
Number of values (total): 1607

Number of families: 1
Number of comparisons per family: 36
Alpha: 0.05

Table S2. Dunn's Post-hoc Test on Duodenal Villi Surface Area Data

Dunn's multiple comparisons test	Mean rank diff.	Significant?	Summary	Adjusted P Value	
No injection vs. H_2O	46.52	No	ns	>0.9999	A-B
No injection vs. Oil 0.5%	120.3	No	ns	0.5196	A-C
No injection vs. ZN	103.6	No	ns	>0.9999	A-D
No injection vs. ZL	-104.9	No	ns	>0.9999	A-E
No injection vs. RN	24.97	No	ns	>0.9999	A-F
No injection vs. RL	-283.5	Yes	****	<0.0001	A-G
No injection vs. ZNRN	-49.31	No	ns	>0.9999	A-H
No injection vs. ZLRL	-219.9	Yes	***	0.0003	A-I
H_2O vs. Oil 0.5%	73.82	No	ns	>0.9999	B-C
H_2O vs. ZN	57.09	No	ns	>0.9999	B-D
H_2O vs. ZL	-151.4	No	ns	0.0771	B-E
H_2O vs. RN	-21.55	No	ns	>0.9999	B-F
H_2O vs. RL	-330	Yes	****	<0.0001	B-G
H_2O vs. ZNRN	-95.83	No	ns	>0.9999	B-H
H_2O vs. ZLRL	-266.5	Yes	****	<0.0001	B-I
Oil 0.5% vs. ZN	-16.72	No	ns	>0.9999	C-D

Oil 0.5% vs. ZL	-225.3	Yes	***	0.0002	C-E
Oil 0.5% vs. RN	-95.37	No	ns	>0.9999	C-F
Oil 0.5% vs. RL	-403.9	Yes	****	<0.0001	C-G
Oil 0.5% vs. ZNRN	-169.6	Yes	*	0.0189	C-H
Oil 0.5% vs. ZLRL	-340.3	Yes	****	<0.0001	C-I
ZN vs. ZL	-208.5	Yes	***	0.0009	D-E
ZN vs. RN	-78.64	No	ns	>0.9999	D-F
ZN vs. RL	-387.1	Yes	****	<0.0001	D-G
ZN vs. ZNRN	-152.9	No	ns	0.0647	D-H
ZN vs. ZLRL	-323.6	Yes	****	<0.0001	D-I
ZL vs. RN	129.9	No	ns	0.3047	E-F
ZL vs. RL	-178.6	Yes	*	0.0106	E-G
ZL vs. ZNRN	55.62	No	ns	>0.9999	E-H
ZL vs. ZLRL	-115	No	ns	0.7225	E-I
RN vs. RL	-308.5	Yes	****	<0.0001	F-G
RN vs. ZNRN	-74.28	No	ns	>0.9999	F-H
RN vs. ZLRL	-244.9	Yes	****	<0.0001	F-I
RL vs. ZNRN	234.2	Yes	****	<0.0001	G-H
RL vs. ZLRL	63.58	No	ns	>0.9999	G-I
ZNRN vs. ZLRL	-170.6	Yes	*	0.0181	H-I
**Test details**	**Mean rank 1**	**Mean rank 2**	**Mean rank diff.**	**n1**	**n2**
No injection vs. H_2O	764	717.5	46.52	176	180
No injection vs. Oil 0.5%	764	643.7	120.3	176	180
No injection vs. ZN	764	660.4	103.6	176	179
No injection vs. ZL	764	869	-104.9	176	174
No injection vs. RN	764	739.1	24.97	176	180

No injection vs. RL	764	1048	-283.5	176	180
No injection vs. ZNRN	764	813.3	-49.31	176	180
No injection vs. ZLRL	764	984	-219.9	176	178
H_2O vs. Oil 0.5%	717.5	643.7	73.82	180	180
H_2O vs. ZN	717.5	660.4	57.09	180	179
H_2O vs. ZL	717.5	869	-151.4	180	174
H_2O vs. RN	717.5	739.1	-21.55	180	180
H_2O vs. RL	717.5	1048	-330	180	180
H_2O vs. ZNRN	717.5	813.3	-95.83	180	180
H_2O vs. ZLRL	717.5	984	-266.5	180	178
Oil 0.5% vs. ZN	643.7	660.4	-16.72	180	179
Oil 0.5% vs. ZL	643.7	869	-225.3	180	174
Oil 0.5% vs. RN	643.7	739.1	-95.37	180	180
Oil 0.5% vs. RL	643.7	1048	-403.9	180	180
Oil 0.5% vs. ZNRN	643.7	813.3	-169.6	180	180
Oil 0.5% vs. ZLRL	643.7	984	-340.3	180	178
ZN vs. ZL	660.4	869	-208.5	179	174
ZN vs. RN	660.4	739.1	-78.64	179	180
ZN vs. RL	660.4	1048	-387.1	179	180
ZN vs. ZNRN	660.4	813.3	-152.9	179	180
ZN vs. ZLRL	660.4	984	-323.6	179	178
ZL vs. RN	869	739.1	129.9	174	180
ZL vs. RL	869	1048	-178.6	174	180
ZL vs. ZNRN	869	813.3	55.62	174	180
ZL vs. ZLRL	869	984	-115	174	178
RN vs. RL	739.1	1048	-308.5	180	180
RN vs. ZNRN	739.1	813.3	-74.28	180	180
RN vs. ZLRL	739.1	984	-244.9	180	178

RL vs. ZNRN	1048	813.3	234.2	180	180
RL vs. ZLRL	1048	984	63.58	180	178
ZNRN vs. ZLRL	813.3	984	-170.6	180	178

3. Duodenal Crypt Depth Kruskal-Wallis Test with Dunn's Post Hoc Test

Kruskal-Wallis test
P value: <0.0001
Exact or approximate P value?: Approximate
P value summary: ****
Do the medians vary significantly (P < 0.05)?: Yes
Number of groups: 9
Kruskal-Wallis statistic: 209.5

Data summary
Number of treatments (columns): 9
Number of values (total): 1614

Number of families: 1
Number of comparisons per family: 36
Alpha: 0.05

Table S3. Dunn's Post-hoc Test on Duodenal Crypt Depth Data

Dunn's multiple comparisons test	Mean rank diff.	Significant?	Summary	Adjusted P Value	
No injection vs. H_2O	-456.8	Yes	****	<0.0001	A-B
No injection vs. Oil 0.5%	-412.4	Yes	****	<0.0001	A-C
No injection vs. ZN	-156.6	No	ns	0.0532	A-D
No injection vs. ZL	-183.6	Yes	**	0.0072	A-E
No injection vs. RN	-132.6	No	ns	0.2621	A-F
No injection vs. RL	-213.1	Yes	***	0.0005	A-G
No injection vs. ZNRN	112.3	No	ns	0.8153	A-H
No injection vs. ZLRL	-216.5	Yes	***	0.0004	A-I
H_2O vs. Oil 0.5%	44.4	No	ns	>0.9999	B-C
H_2O vs. ZN	300.2	Yes	****	<0.0001	B-D
H_2O vs. ZL	273.2	Yes	****	<0.0001	B-E
H_2O vs. RN	324.2	Yes	****	<0.0001	B-F
H_2O vs. RL	243.6	Yes	****	<0.0001	B-G
H_2O vs. ZNRN	569.1	Yes	****	<0.0001	B-H
H_2O vs. ZLRL	240.3	Yes	****	<0.0001	B-I

Oil 0.5% vs. ZN	255.8	Yes	****	<0.0001	C-D
Oil 0.5% vs. ZL	228.8	Yes	***	0.0001	C-E
Oil 0.5% vs. RN	279.8	Yes	****	<0.0001	C-F
Oil 0.5% vs. RL	199.2	Yes	**	0.0018	C-G
Oil 0.5% vs. ZNRN	524.7	Yes	****	<0.0001	C-H
Oil 0.5% vs. ZLRL	195.9	Yes	**	0.0025	C-I
ZN vs. ZL	-26.94	No	ns	>0.9999	D-E
ZN vs. RN	24.03	No	ns	>0.9999	D-F
ZN vs. RL	-56.52	No	ns	>0.9999	D-G
ZN vs. ZNRN	268.9	Yes	****	<0.0001	D-H
ZN vs. ZLRL	-59.87	No	ns	>0.9999	D-I
ZL vs. RN	50.97	No	ns	>0.9999	E-F
ZL vs. RL	-29.58	No	ns	>0.9999	E-G
ZL vs. ZNRN	295.8	Yes	****	<0.0001	E-H
ZL vs. ZLRL	-32.93	No	ns	>0.9999	E-I
RN vs. RL	-80.55	No	ns	>0.9999	F-G
RN vs. ZNRN	244.9	Yes	****	<0.0001	F-H
RN vs. ZLRL	-83.9	No	ns	>0.9999	F-I
RL vs. ZNRN	325.4	Yes	****	<0.0001	G-H
RL vs. ZLRL	-3.35	No	ns	>0.9999	G-I
ZNRN vs. ZLRL	-328.8	Yes	****	<0.0001	H-I
**Test details**	**Mean rank 1**	**Mean rank 2**	**Mean rank diff.**	**n1**	**n2**
No injection vs. H_2O	623	1080	-456.8	178	180
No injection vs. Oil 0.5%	623	1035	-412.4	178	179
No injection vs. ZN	623	779.6	-156.6	178	180
No injection vs. ZL	623	806.5	-183.6	178	179

No injection vs. RN	623	755.6	-132.6	178	178
No injection vs. RL	623	836.1	-213.1	178	180
No injection vs. ZNRN	623	510.7	112.3	178	180
No injection vs. ZLRL	623	839.5	-216.5	178	180
H_2O vs. Oil 0.5%	1080	1035	44.4	180	179
H_2O vs. ZN	1080	779.6	300.2	180	180
H_2O vs. ZL	1080	806.5	273.2	180	179
H_2O vs. RN	1080	755.6	324.2	180	178
H_2O vs. RL	1080	836.1	243.6	180	180
H_2O vs. ZNRN	1080	510.7	569.1	180	180
H_2O vs. ZLRL	1080	839.5	240.3	180	180
Oil 0.5% vs. ZN	1035	779.6	255.8	179	180
Oil 0.5% vs. ZL	1035	806.5	228.8	179	179
Oil 0.5% vs. RN	1035	755.6	279.8	179	178
Oil 0.5% vs. RL	1035	836.1	199.2	179	180
Oil 0.5% vs. ZNRN	1035	510.7	524.7	179	180
Oil 0.5% vs. ZLRL	1035	839.5	195.9	179	180
ZN vs. ZL	779.6	806.5	-26.94	180	179
ZN vs. RN	779.6	755.6	24.03	180	178
ZN vs. RL	779.6	836.1	-56.52	180	180
ZN vs. ZNRN	779.6	510.7	268.9	180	180
ZN vs. ZLRL	779.6	839.5	-59.87	180	180
ZL vs. RN	806.5	755.6	50.97	179	178
ZL vs. RL	806.5	836.1	-29.58	179	180
ZL vs. ZNRN	806.5	510.7	295.8	179	180
ZL vs. ZLRL	806.5	839.5	-32.93	179	180
RN vs. RL	755.6	836.1	-80.55	178	180
RN vs. ZNRN	755.6	510.7	244.9	178	180

RN vs. ZLRL	755.6	839.5	-83.9	178	180
RL vs. ZNRN	836.1	510.7	325.4	180	180
RL vs. ZLRL	836.1	839.5	-3.35	180	180
ZNRN vs. ZLRL	510.7	839.5	-328.8	180	180

4. Duodenal Morphology

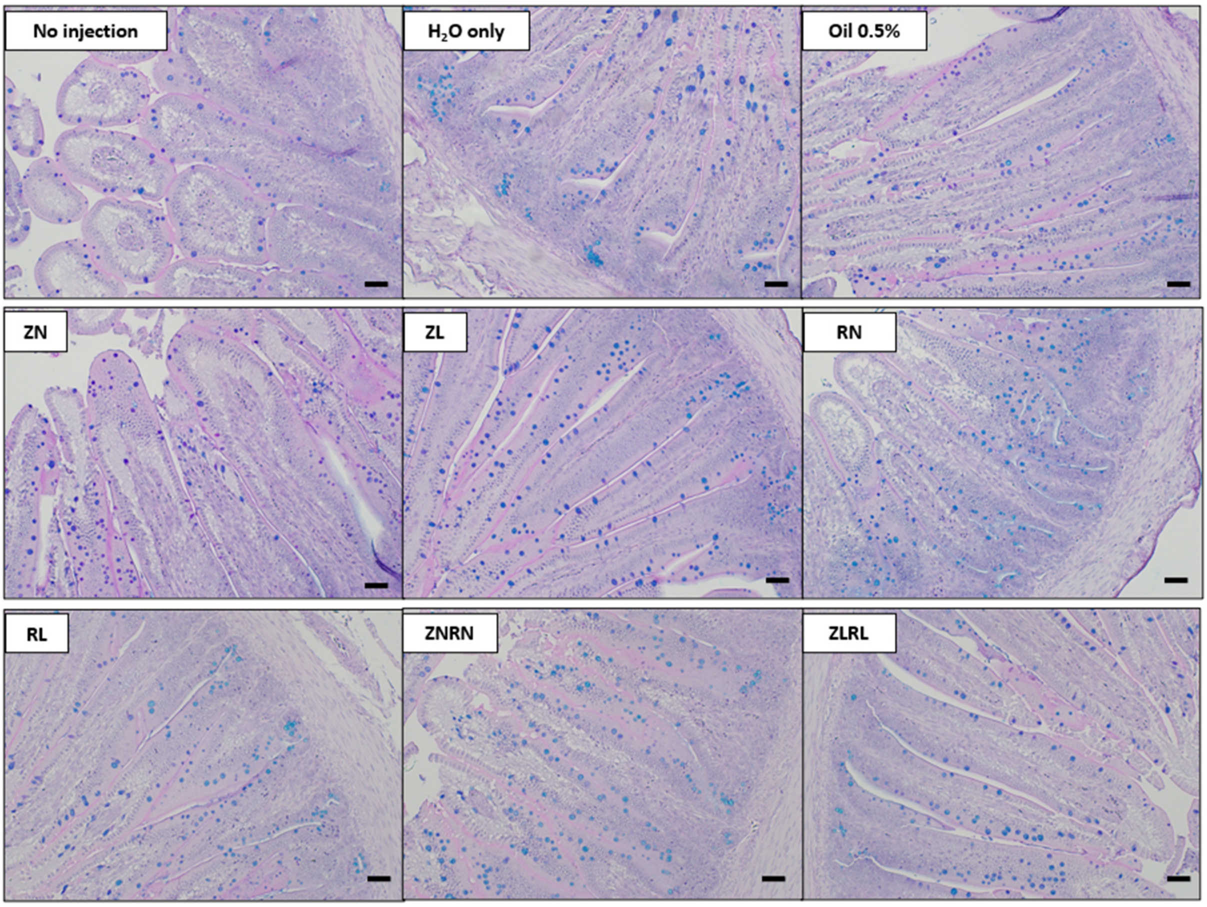

Figure S1. Representative images of duodenal morphology per treatment group. Alcian Blue/Periodic acid-Schiff stain is shown. Bar = 20 μM
